# A subduction and mantle plume origin for Samoan volcanism

**DOI:** 10.1038/s41598-018-28267-3

**Published:** 2018-07-11

**Authors:** Vincent Strak, Wouter P. Schellart

**Affiliations:** 1 School of Earth, Atmosphere and Environment, Monash University, Melbourne, VIC 3800 Australia; 2 Department of Earth Sciences, Vrije Universiteit Amsterdam, Amsterdam, Netherlands; 3 Instituto Dom Luiz, Lisbon University, Lisbon, Portugal

## Abstract

The origin of Samoan volcanism in the southwest Pacific remains enigmatic. Whether mantle melting is solely caused by a mantle plume is questionable because some volcanism, here referred to as non-hotspot volcanism, defies the plume model and its linear age-progression trend. Indeed, non-hotspot volcanism occurred as far as 740 km west of the predicted Samoan hotspot after 5 Ma. Here we use fully-dynamic laboratory subduction models and a tectonic reconstruction to show that the nearby Tonga-Kermadec-Hikurangi (TKH) subduction zone induces a broad mantle upwelling around the northern slab edge that coincides with the non-hotspot volcanic activity after 5 Ma. Using published potential mantle temperatures for the ambient mantle and Samoan mantle plume, we find that two geodynamic processes can explain mantle melting responsible for intraplate volcanism in the Samoan region. We propose that before 5 Ma, the volcanism is consistent with the plume model, whereas afterwards non-hotspot volcanism resulted from interaction between the Subduction-Induced Mantle Upwelling (SIMU) and Samoan mantle plume material that propagated west from the hotspot due to the toroidal component of slab rollback-induced mantle flow. In this geodynamic scenario, the SIMU drives decompression melting in the westward-swept plume material, thus producing the non-hotpot volcanism.

## Introduction

**Figure 1:**
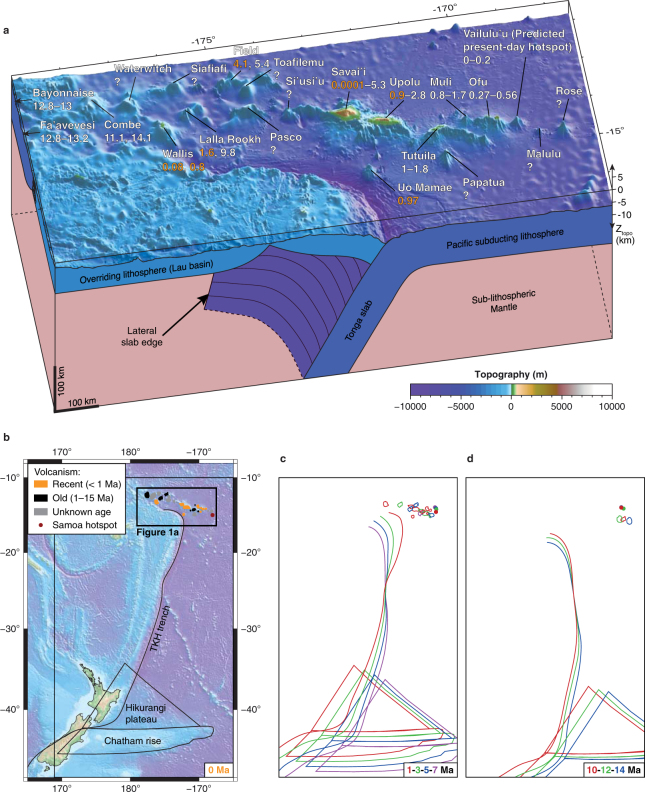
Unique tectonic setting and kinematics of the Samoan volcanic province, close to the Tonga-Kermadec-Hikurangi (TKH) subduction zone. (**a**) Perspective bathymetry and line drawing showing Samoan volcanoes with their name and northern slab edge of the TKH subduction zone. Numbers indicate known age of volcanism. Orange text highlights ages too young to fit the plume model. (**b**) Present-day bathymetry and main tectonic features of the TKH subduction zone. (**c**,**d**) Tectonic reconstruction depicting evolution of the TKH trench, the position of Samoan volcanism and of the Hikurangi Plateau and Chatham Rise at 1, 3, 5, 7 Ma (**c**) and 10, 12, 14 Ma (**d**). The topographic maps in Fig. 1a,b were created using the GEBCO_2014 Grid (version 20150318, www.gebco.net) with 0.5 arc-minute resolution and all maps were plotted using the GMT software developed by Wessel and Smith (1991)^60^ (version 4.5.14, http://gmt.soest.hawaii.edu). The drawings in tectonic reconstruction maps of Fig. 1b,c,d were done using the GPlates software (version 2.0, http://www.gplates.org). A data bundle provided with the software allowed us to retrace the evolution of the TKH trench, the Pacific subducting plate, the Samoan volcanoes, the Hikurangi Plateau, and the Chatham rise^52–55^, as well as the Samoan hotspot^1,56–58^, using the global rotation model of Matthews *et al*.^52^ and the Indo-Atlantic moving hotspot reference frame as a reference^59^.

The quasi linear distribution of the Samoan volcanoes over ∼1,300 km seems to indicate a deep mantle plume for their origin (Fig. 1a). Tomography models moreover provide images of a low-velocity seismic structure in the mantle that could represent a plume originating from the core-mantle boundary below the Samoan islands^1–3^. Such a deep mantle plume could produce a localised hotspot from which a volcano periodically develops at the surface of the moving Pacific lithosphere, thereby producing a linear, time-progressive, westerly ageing volcanic chain^4^. In this geodynamic concept, the most recent and most productive volcanic activity located at Vailulu’u would indicate the present-day location of the Samoan hotspot^5^ (Fig. 1a). In addition to the seismic studies and the geometric alignment of the volcanoes, a number of geochemistry studies are in favour of the plume model^5–8^. However, the plume model also faces significant contradictions, most notably the recent occurrence of volcanism throughout the chain, which calls for an alternative mechanism to explain the rejuvenated volcanism in the Samoan region.

A significant limit to the plume model is revealed in the western Samoan region where several volcanoes have experienced volcanic activity that is too young to fit the predicted age^9^ (Fig. 1a). In particular, 40Ar/39Ar and K/Ar dating of volcanic rocks on Field, Lalla Rookh and Wallis volcanoes returned ages as young as 4.1 Ma, 1.6 Ma, and 0.08 Ma, respectively^5,8^. These ages plot well away, by ∼2–11 Myrs, from the age-distance relationship predicted by the plume model regardless of the plate velocity model or absolute plate motion model assumed. Such young ages may not indicate the shield stage, the initial construction phase of volcanoes, but rather rejuvenation of the volcanic activity on a shield volcano. In addition, other volcanoes of unknown age, including Bayonnaise, Fa’avevisi, Combe, Waterwitch, Pasco, Toafilemu, and Papatua show a geochemical signature that is characteristic of the dated rejuvenated lavas^7,8^. Dating these volcanoes could possibly give young rejuvenation ages as well, and it is questionable if they are reconcilable with a plume model. Further, in the eastern Samoan region, volcanoes located closer to the predicted hotspot, though still at a significant distance of more than 300 km to the west, have also been active recently. Two striking examples are Savai’i and Upolu, which both produced voluminous amounts of lava after the shield stage in comparison, for example, with late stage volcanism in Hawaii^8^. In particular, the island of Savai’i has a shield age of ∼5 Ma^6^ but has undergone rejuvenated volcanism until very recently, the last known eruption being in 1911^8^. Known processes of post-erosional volcanism as observed in classical plume-induced volcanoes such as in Hawaii are unlikely to explain the rejuvenated volcanism west of Savai’i because in Hawaii it ceases at most ∼5 Myrs after the shield volcano is born^10^. Hence, an alternative mechanism to the plume model is required to explain the abnormally young volcanism in the western Samoan region, and possibly the voluminous rejuvenated volcanism on Savai’i and Upolu. To complicate matters further regarding the volcano age distribution, Malulu and Rose, two volcanoes located east of the predicted present-day hotspot, are thought to be older^8^ whereas the plume model predicts that these volcanoes should not exist yet but only be built in the future or recently if one considers the relative proximity with the present-day hotspot.

Another striking feature of the Samoan region relates to the spatial distribution of volcanoes of which some tend to break the linear trend (Fig. 1a). For example, Uo Mamae and Papatua are clearly offset 120 km south of Upolu and 60 km south of Tutuila, respectively. Interestingly, Uo Mamae experienced volcanic activity at 0.97 Ma^11^, similar to the late rejuvenated volcanism on Upolu. The western Samoan region also seems less linear than the well aligned en-échelon volcanic trails of the eastern Samoan region^8^. Moreover, a particularity of the Samoan volcanism is that in general the volcanic rocks of rejuvenated origin have a distinctive isotopic geochemistry when compared with the shield lavas^9,11^. This was an additional source of motivation that led to the proposal of alternative mechanisms to explain the abnormal volcanic rejuvenation throughout the chain. Suggested mechanisms include multiple consecutive mantle plume sources^7^, and lithospheric flexure and cracking due to the nearby TKH subduction zone in combination with shear melting at the base of the lithosphere^8,9,11^. Proximity of the Samoan region to the northern termination of the TKH subduction zone (Fig. 1b) indeed suggests that subduction related processes could provide an alternative origin for the Samoan volcanism. A first examination of our tectonic reconstruction (see Methods) shows a phase of slab rollback starting at 10 Ma and accelerating at 5 Ma, in remarkable concomitance with the rejuvenation of volcanism throughout the volcanic chain after 5 Ma (Fig. 1c,d).

**Figure 2:**
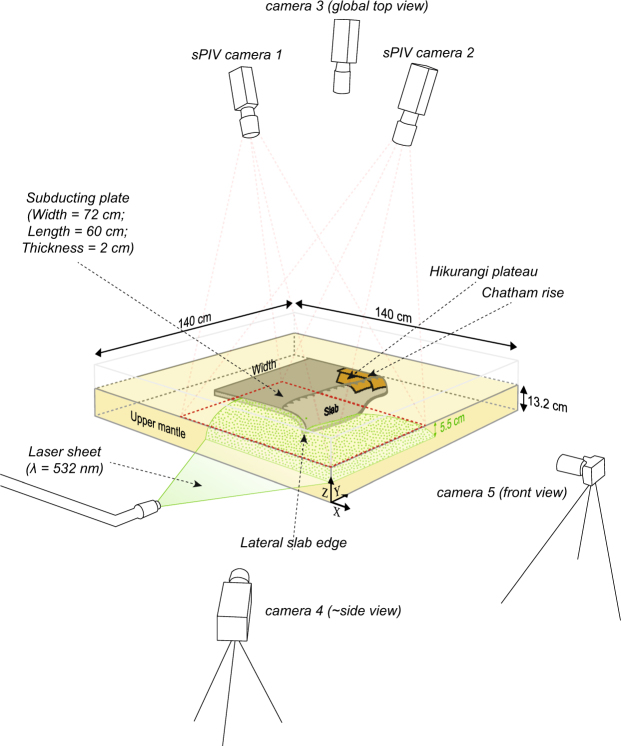
Experimental setup with the subducting plate depicted during the slab free sinking phase of the subduction process. The scaling with the natural prototype is done by ensuring that dynamic similarity is respected (see Methods for details). sPIV cameras 1 and 2 were placed in stereoscopic arrangement above the model and recorded pictures during intermittent laser sheet emission, which illuminated phosphorescent particles at 5.5 cm (275 km) depth inside the glucose syrup that simulates the sublithospheric upper mantle. Cameras 3, 4 and 5 were fixed and dedicated to study the subducting plate kinematics. The 3 components of the mantle flow velocity field on a plane could be calculated using a stereo cross-correlation technique. This figure is modified from Strak and Schellart^23^.

Quasi-toroidal mantle return flow driven by slab rollback is known to occur around the lateral slab edges of subduction zones^12–15^, including around the northern edge of the Tonga slab^16,17^. This return flow has a component of upwelling that is produced away from the lateral slab edges in the trench parallel direction, thus in an intraplate setting^18–20^. The TKH subduction zone is unique because it is characterised by high trench retreat velocities in the north, reaching 8.2 cm/yr averaged over the last three Myrs as indicated by our kinematic reconstruction, that progressively decrease to the south and reverse to trench advance (Fig. 1b). This asymmetry in trench retreat reflects an along-trench gradient in slab sinking and rollback, which coincides with faster trenchward subducting plate motion, leading to more slab material being consumed in the north as seen on tomographic images^21,22^. Mantle flow that develops in such a subduction setting may differ from the more common symmetrical trench retreat cases. We thus developed fully-dynamic, laboratory-based, subduction models of the TKH subduction zone to simulate asymmetric subduction and to quantify the subduction-induced mantle flow and associated upwelling (SIMU) around the northern lateral slab edge (see Methods and Fig. 2). In our models the kinematic asymmetry is caused by presence of the positively buoyant Hikurangi plateau and Chatham rise born by the Pacific plate in the south of the TKH subduction zone. Because they are positively buoyant, the Hikurangi plateau and Chatham rise resist slab sinking and rollback whereas further north the Tonga-Kermadec slab segment quickly migrates eastward since there is no other resistance to slab motion than viscous mantle drag. We moreover performed a tectonic reconstruction (see Methods) to compare with our modelling results, allowing us to test whether Samoan volcanism can possibly be related to the SIMU occurring north of the Tonga slab.

## Results

### Geodynamic modelling of Subduction-Induced Mantle Upwelling (SIMU)

**Figure 3:**
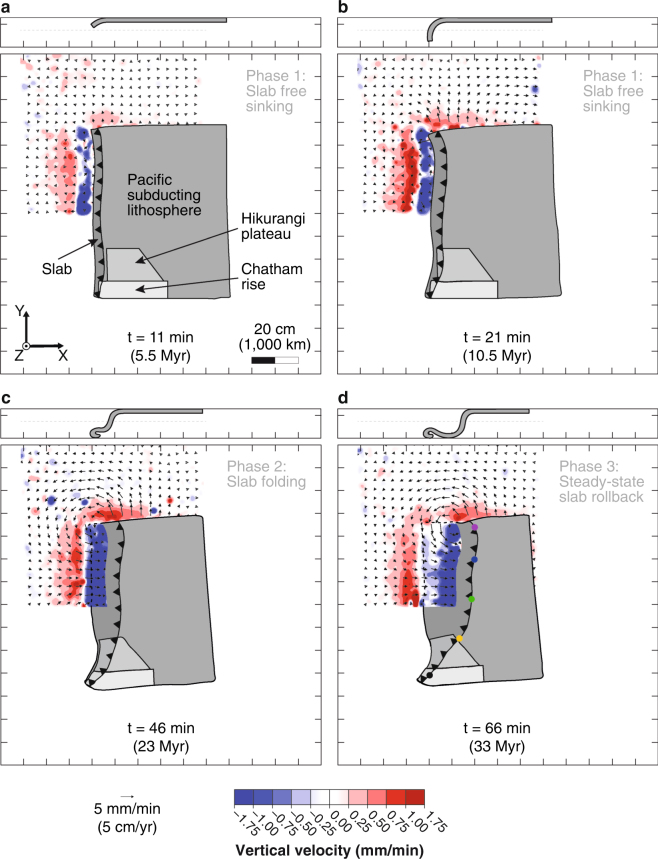
Predicted SIMU position and magnitude from laboratory subduction model. The four panels (a–d) show the position of the plate, slab, and trench (line drawing) at different stages of the subduction process with: (**a**,**b**) the first phase of free slab sinking; (**c**) the end of the second phase of slab folding at the rigid bottom boundary that simulates the 660 km mantle discontinuity; (**d**) the third phase of steady-state slab rollback. On each panel we show the three components of the computed mantle flow velocity field at 5.5 cm (275 km) depth in map view (bottom), and cross-sectional slab geometry at the northern edge (representing the northern Tonga subduction zone edge) (top).

**Figure 4:**
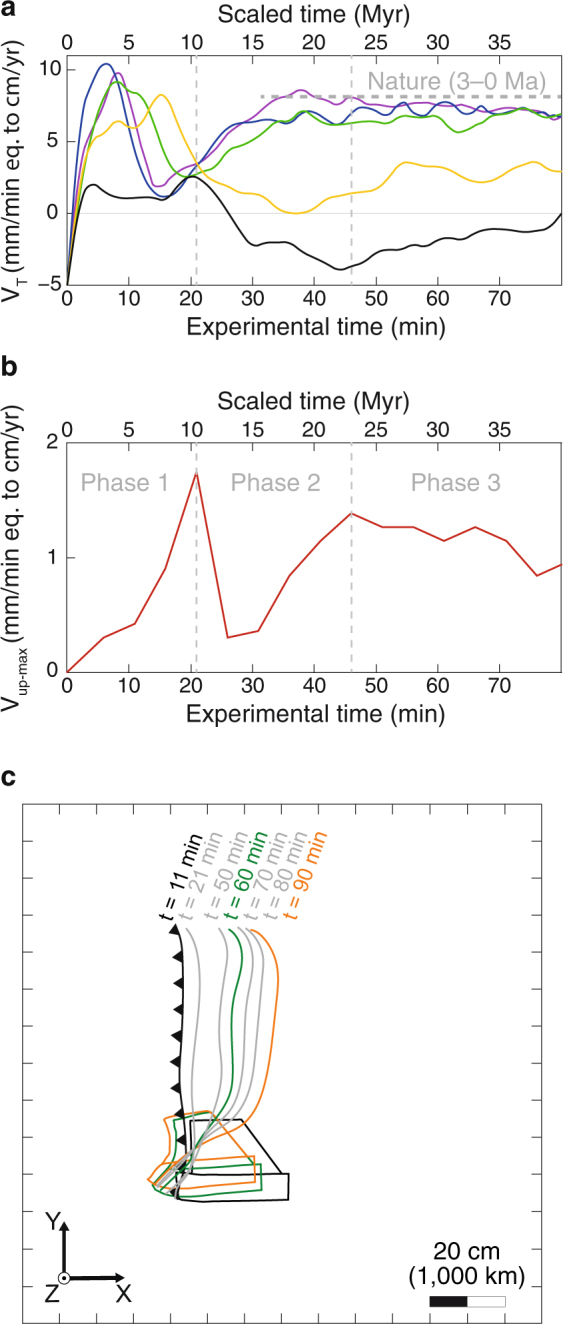
Predicted evolution of trench kinematics and SIMU magnitude. (**a**) Evolution of trench retreat velocity in time for five locations along the trench, reported on Fig. 3d. (**b**) Evolution of SIMU maximum velocity in time. (**c**) Line drawing showing the evolution of asymmetric trench kinematics.

Out of 10 subduction experiments that were performed, we report here the results of one model (Figs 3, 4 and Table S1) that best reproduces the kinematics of trench motion as determined by our tectonic reconstruction, and best approximates the present-day slab geometry in the upper mantle. The experiment evolves in three subduction phases, as commonly observed in fully-dynamic subduction models, as a result of the interaction between the slab and upper-lower mantle transition zone simulated by the rigid bottom of the tank^13,15^ (Fig. 3). The three subduction phases are as follows: (1) An initial slab free sinking phase with progressive acceleration of the subduction process due to increasing volume of negatively buoyant slab material diving in the relatively less dense upper mantle; (2) A slab folding phase due to interaction with the rigid bottom of the tank simulating the 660 km mantle discontinuity; (3) A steady-state slab rollback phase during which velocities become approximately constant over time. During the steady-state rollback phase of our model (phase 3 on Figs 3d and 4a,b), the trench retreats quickly in the north, with scaled velocities reaching 8 cm/yr, and it progressively slows down to the south to reverse to trench advance in the middle of the Hikurangi plateau (Fig. 4c). This trench kinematics is indeed similar to the natural case (Figs 1c,d and 4a). A simplification of our model is the absence of a lower mantle. Despite this simplification, the similarity in trench kinematics indicates a good reproduction of slab sinking and lateral migration in the upper mantle.

In our model, the fast slab rollback in the north produces a quasi-toroidal upper mantle flow around the northern slab edge, with a component of upwelling (Fig. 3). The SIMU is broad, initiates soon after subduction inception and is always present during the subduction process (Figs 3 and 4b). Variations in upwelling rate correspond to the 3 subduction phases (Fig. 4b). We note that the SIMU occurs both outboard of the sub-slab and mantle wedge domain (Fig. 3c,d) whereas in models with uniform trench retreat it is predominantly focused outboard of the sub-slab domain^23^. The asymmetric slab rollback of the TKH subduction zone thus promotes the development of a broad SIMU extending perpendicularly to the trench, with scaled upward velocities averaging 1.2 cm/yr during the steady-state slab rollback phase.

### Comparison with tectonic reconstruction

**Figure 5:**
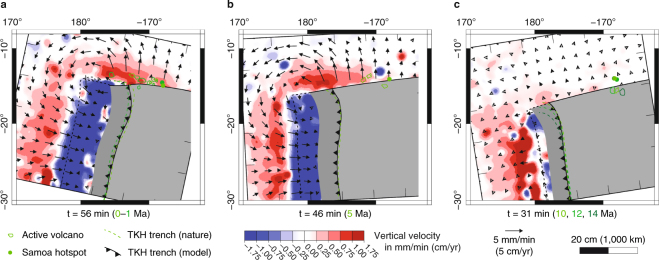
Correlation between Samoan volcanism and predicted SIMU. Overlays of kinematic reconstruction with subduction model mantle flow velocity field computed at 5.5 cm (275 km) depth showing that: (**a**) SIMU superimposes very well with Samoan volcanism at 0 and 1 Ma; (**b**) The correlation possibly starts at 5 Ma; (**c**) Before 10 Ma the SIMU is minimal due to slow slab rollback and the separation between the lateral slab edge and the predicted Samoan hotspot location is large, thus the interaction between SIMU and plume material cannot explain the volcanism.

We compare our model with the last 14 Myr of the natural prototype kinematics. We use the slab folding phase (phase 2) of our model as an approximation of the subduction dynamics that occurred between 14–10 Ma because it replicates the dominance of down-dip slab motion over minimal slab rollback (Figs 1d and 5c). We then use the steady-state phase (phase 3) of our model as it replicates well the subduction dynamics in nature between 10–0 Ma where slab rollback becomes significant (Figs 1c and 5a,b).

We find that the broad SIMU of our subduction model overlaps with all intraplate volcanoes in the Samoa region that are active at 0–1 Ma (Fig. 5a). In particular, areas of high SIMU velocities match very well with volcanoes that were active recently despite their location away from the predicted hotspot, such as Savai’i at 0 Ma and Wallis at ∼1 Ma. The correlation starts to be less clear at 5 Ma (Fig. 5b) and the SIMU is of low magnitude for older times due to very slow slab rollback (Fig. 1d), which thus compares with the slow trench retreat observed during phase 2 of our model (Fig. 5c). These results strongly suggest that the SIMU around the northern slab edge of the TKH subduction zone contributes to causing the intraplate volcanism in the Samoan region after 5 Ma, in particular the rejuvenated volcanism occurring west of the predicted hotspot. The location of the Samoan hotspot after 5 Ma is remarkably distant from the active non-hotspot volcanoes, but it can explain older Samoan volcanism (Fig. 5c).

## Discussion

**Figure 6:**
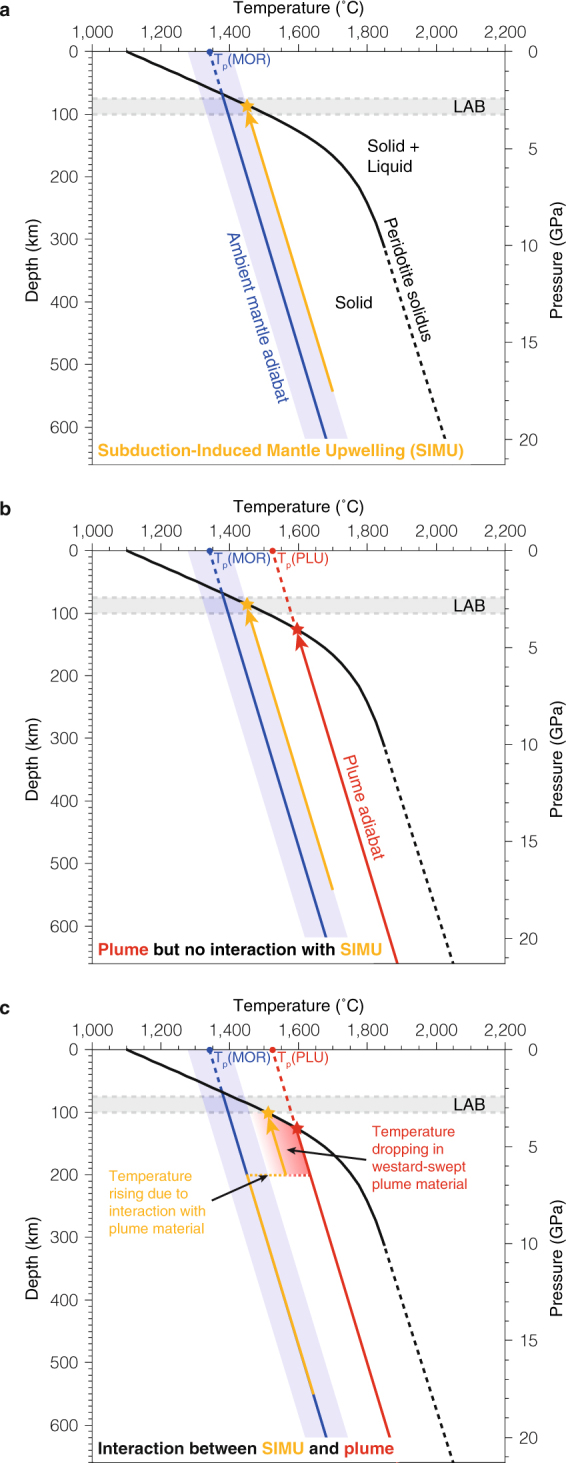
Predictions for onset of mantle melting. Calculations to explain mantle melting by: (**a**) SIMU only; (**b**) Samoan plume only (no interaction with SIMU); (**c**) SIMU interacting with Samoan plume. In (**c**) the onset of mantle melting is facilitated because the SIMU drives decompression melting in the westward-swept Samoan plume material, thus producing the non-hotpot volcanism occurring west of the Samoan hotspot after 5 Ma.

The cause for mantle melting that ultimately leads to formation of Samoan volcanism can be discussed in light of previous research on potential temperature *T*_P_ for the ambient mantle and mantle plumes. *T*_P_ is the extrapolation at the Earth’s surface of adiabatic temperature-depth profiles, and is determined from the primary melt calculated using forward thermodynamic modelling of adiabatic decompression melting^24,25^. Using the intersection between the peridotite solidus curve and the adiabatic temperature profile at a given *T*_P_ , one can assess the depth for the onset of mantle melting in the Samoan region. We compute this mantle melting onset depth that corresponds to the primary melt depth and we use it as a proxy to evaluate the efficiency of three different geodynamic scenarios in producing mantle melting (Fig. 6). We assume a peridotite solidus curve and an adiabatic temperature gradient as determined in previous research^24^, and we use estimated lower and upper bounds of 75 km and 100 km^26^ for the depth of the lithosphere-asthenosphere boundary (LAB) below the Samoan region. We use a *T*_p_ of 1340 ± 60 °C for the ambient mantle (*T*_P_(MOR) in Fig. 6a ) as constrained from estimates at mid-oceanic ridges^27^ since decompression melting there results from passive upwelling with no initial temperature anomaly. In contrast, *T*_P_ estimates for the Samoan mantle plume (*T*_P_(PLU) in Fig. 6b,c) range between 1395 °C and 1524 °C^28^. As can be expected, the Samoan mantle plume is hotter than the surrounding ambient mantle, and the excess temperature is in agreement with *T*_P_ estimates for other plumes^29^. We assume an adiabatic gradient of 0.55 °C/km, which is an average of the values ranging between 0.45–0.7 °C/km that are commonly used in thermodynamic studies^24,30^. Calculation of the dry peridotite solidus is done using the following equation, which was determined experimentally^24^:

1 $${\rm{P}}({{\rm{T}}}_{{\rm{S}}})=\frac{{{\rm{T}}}_{{\rm{S}}}-{{\rm{T}}}_{{\rm{S}}}^{0}}{136}+4.968\times {10}^{-4}{{\rm{e}}}^{(1.2\times {10}^{-2}({{\rm{T}}}_{{\rm{S}}}-{{\rm{T}}}_{{\rm{S}}}^{0}))}$$

with ${\rm{P}}({{\rm{T}}}_{{\rm{S}}})$ the pressure in GPa at a given temperature ${{\rm{T}}}_{{\rm{S}}}$ in °C, and ${{\rm{T}}}_{{\rm{S}}}^{0}$ the extrapolation of temperature at the surface, which is 1,100 °C. We consider a dry peridotite solidus because the Samoan volcanism is located away from the arc volcanism, thus hydration processes should be minimum. In contrast, we infer that wet peridotite solidi should be considered to study the adakite-like volcanism that occurs as a result of interaction between mantle upwelling and slab possibly carrying sediments. Such volcanism does not occur in the Samoan region but does occur in the mantle wedge close to the northern slab edge of the TKH subduction zone^31^.

The process of SIMU (Fig. 6a) occurs in the ambient mantle, hence with the lowest *T*_P_ without bringing any thermal anomaly. The onset of mantle decompression melting in the asthenosphere is possible via this process only if we consider minimum values for the LAB and maximum values for *T*_P_ (orange arrow and star in Fig. 6a). In sharp contrast, the Samoan mantle plume (Fig. 6b) brings a heat excess that allows the onset of melting in the asthenosphere below the LAB, and thus consistently explains the origin of intraplate volcanism occurring near the predicted hotspot. We propose that before 5 Ma, the Samoan mantle plume is the only process responsible for the intraplate volcanism because our tectonic reconstruction shows that volcanic activity always occurred near the predicted hotspot location and far from the northern Tonga subduction segment (Figs 1d and 5c). The plume might also continue to produce Samoan volcanism near the predicted hotspot location after 5 Ma.

To explain the non-hotspot volcanism occurring west of the hotspot after 5 Ma (Fig. 1c), we suggest that the SIMU interacts with Samoan mantle plume material to promote decompression melting (Fig. 6c). Noting that active non-hotspot volcanism became progressively more distant west of the predicted Samoan hotspot between 1–5 Ma (Figs 1c,d and 5a,b), we propose that Samoan mantle plume material has been progressively dragged west of the hotspot after 5 Ma by the toroidal component of subduction-induced mantle flow. Toroidal return flow has already been suggested around the northern slab edge of the Tonga slab by azimuthal anisotropy^17^, consistently with trench-parallel anisotropic fast directions in the sub-slab domain^32^. Such toroidal mantle flow is known to strongly deform the mantle plume tail and head^33^, and it has been suggested for the Samoan plume using geochemistry and modelling^16,31,34^. Variations of the isotropic shear velocity at 250 km depth north of the Tonga slab are also in agreement with the suggestion that hot material from the Samoan plume is swept westward^35,36^. Considering the onset time of the interaction between the subduction-induced toroidal component of mantle flow and the Samoan plume at around 5 Ma, the propagated plume material may have originated from the Samoan plume head that may have been present at that time. Alternatively, the dragged plume material could come from plume tail material ponded underneath the base of the lithosphere. Our geodynamic subduction model demonstrates that the rollback-induced return flow around the northern edge of the Tonga slab has a strong upwelling component over a broad area. We propose that this SIMU is the driver of decompression melting in the hot westward-swept Samoan plume material after 5 Ma.

Our two-phase geodynamic scenario reconciles a range of observations and geochemical data. It satisfactorily explains the occurrence of intraplate volcanism due to the Samoan hotspot at all times and also of non-hotspot volcanism west of the hotspot after 5 Ma. It is moreover consistent with the geochemistry of shield and rejuvenated lavas from the regions that have a common Samoan mantle plume source^5^ despite the great distance of the rejuvenated lavas from the hotspot. It also accounts for the different isotopic signatures of the shield (hotspot) and rejuvenated (non-hotspot) lavas that could thus be due to the two different mechanisms triggering decompression melting in the upper mantle. The triggering of decompression melting in the westward-swept Samoan plume material by SIMU provides a new alternative origin to intraplate volcanism that can be explored in other comparable tectonic settings. An interesting parallel can be done with the triggering of decompression melting in the Hawaiian plume head by upwellings resulting from small-scale lithospheric convection that might produce volcanism that is too young to be attributed to the Hawaiian hotspot^37^. Another type of interaction that has been proposed is between mantle plume, mid-oceanic ridges and background mantle flow to explain volcanism occurring far away from the Réunion^38^ and Tristan Da Cunha^39^ hotspots. In this case, decompression melting occurs near the mid-oceanic ridges where the oceanic lithosphere is thinner.

## Methods

### Fully-dynamic laboratory subduction models

The laboratory modelling builds on previous studies that use a fully-dynamic approach to study the subduction process^13,20,23,40,41^. The models are self-consistent and driven by buoyancy forces only, except that we initiate subduction by creating a small slab perturbation of about 3 cm, scaling to 150 km, at the beginning of the experiments. The driving force is the slab negative buoyancy and the resistive forces are assumed to be dominated by viscous resistance and drag in the ambient mantle, slab, and lithospheric plate. The fully-dynamic (buoyancy-driven) modelling approach allows us to investigate the mantle flow that results solely from the dynamics of slab sinking and rollback. In this approach, the rheological response of the lithosphere and asthenosphere is assumed to be dominantly viscous over geological timescales. Our scaling procedure follows previous subduction modelling studies that use the Stokes’ settling law to describe the velocity of the sinking slab^40,42,43^, defined as follows:

2 $$v \sim C\frac{{\rm{\Delta }}\rho {l}^{2}g}{\eta }$$

where $v$ is the slab sinking velocity, $C$ is a constant, ${\rm{\Delta }}\rho$ is the density contrast between the slab and the ambient mantle, $l$ is a characteristic length, $g$ is the gravitational acceleration, and $\eta$ is the dynamic shear viscosity of the sublithospheric upper mantle. To achieve dynamic similarity our scaling uses identical force balance between model (subscript $m$) and natural prototype (subscript $p$), such that:

3 $$\frac{{\rm{\Delta }}{\rho }_{m}{l}_{m}^{2}\,{g}_{m}}{{\eta }_{m}{v}_{m}}=\frac{{\rm{\Delta }}{\rho }_{p}{l}_{p}^{2}{g}_{p}}{{\eta }_{p}{v}_{p}}$$

which can be rewritten as follows:

4 $$\frac{{\eta }_{m}}{{\eta }_{p}}=\frac{{\rm{\Delta }}{\rho }_{m}{l}_{m}{t}_{m}}{{\rm{\Delta }}{\rho }_{p}{l}_{p}{t}_{p}}$$

We use a length scale ratio *L*_*m*_/*L*_*p*_ of 2.0 × 10^−7^ (1 cm in the models represents 50 km in nature), a time scale ratio *t*_*m*_/*t*_*p*_ of 3.81 × 10^−12^ (1 min in the models scales to 0.5 Ma), a glucose syrup (sublithospheric upper mantle equivalent) viscosity ${\eta }_{m}$ of 202 ± 8 Pa·s at 20 °C, and we scale our models using density contrasts in relation to the upper mantle^41^, with a density of 3,200 kg^·^m^−3^ for the upper mantle in nature, a density of 1,420 kg^·^m^−3^ for the glucose syrup in the models, and density contrasts between the slab and mantle of 80 kg^·^m^−3^ in nature and 97 kg^·^m^−3^ in the models (see details in paragraph below). With these numbers, we calculate a sublithospheric upper mantle viscosity ${\eta }_{p}$ of ~2.19 × 10^20^ Pa·s, which is in the range of natural estimates of 10^19^–10^21^ ^44^. Another criterion that allows us to achieve dynamic similarity is the use of a small Reynolds number, estimated between 7.03 × 10^−6^ and 2.53 × 10^−4^. Such a small Reynolds number ensures that the models are in the laminar symmetrical flow regime and guarantee the dominance of viscous resistive forces over negligible inertial forces^43^.

The models involve four linear-viscous (Newtonian) layers contained in a rectangular tank that is 140 cm long and 140 cm wide (see Fig. 2). One layer is made of transparent glucose syrup and fills the tank up to 13.2 cm to simulate the sub-lithospheric upper mantle, the rigid bottom of the tank approximating the 660-km discontinuity. The glucose syrup has a density of 1,420 kg·m^−3^ and a viscosity of 202 ± 8 Pa·s at 20 °C. Three layers allow to simulate the Pacific subducting lithosphere that carries the Hikurangi plateau and the Chatham rise to the south of the TKH subduction zone. A 100-km-thick subducting oceanic plate is simulated using a 2-cm-thick layer of silicone (Wacker silicone, from Dow Corning company) mixed with fine iron powder. A 100-km-thick plate is a good first order approximation of the Pacific subducting plate that shows variation in age along the TKH trench, with ages of ∼85 Ma near the Osbourne Trough that increase in both directions to reach ∼125 Ma at the southern termination and ∼110 Ma at the northern termination of the subduction zone^45^. At these ages, 100 km is an upper bound for the depth of the lithosphere-asthenosphere boundary (LAB) because of seafloor flattening at ages greater than 70 Ma^26,46^. Moreover, for oceanic lithosphere older than 80 Ma, geophysical estimates of the Gutenberg discontinuity seem to indicate a LAB at 75 km on average^26^. The subducting plate layer is 72 cm (scaling to 3,600 km) in the trench-parallel direction and 60 cm (scaling to 3,000 km) in the trench-normal direction, leaving a distance of 34 cm between either lateral edge of the slab and the sidewalls to minimise sidewall effects^23^. It has a density of 1517 kg·m^−3^ and a viscosity of 6.32 ± 0.1 × 10^4^ Pa·s, as measured using an Anton Paar Physica MCR301 rheometer. The subducting plate-upper mantle density contrast of 97 kg·m^−3^ reflects natural conditions of a 100-km-thick oceanic lithosphere^47^, although it is slightly higher in the models (17 kg·m^−3^) to negate surface tension effects that are negligible in nature, and it makes the subducting plate negatively buoyant^23^. Both the trailing edge and the lateral edges of the subducting plate are free, representing a mid-oceanic ridge and strike-slip faults, respectively, that offer negligible resistance to subducting oceanic plate motion.

The Hikurangi plateau, an oceanic plateau formed of mafic igneous crust^48^, is simulated using a mixture of Wacker silicone and Dow Corning 3176 Dilatant compound (referred to as pink putty) in weight proportion of 10.66% and 89.34%, respectively. The Wacker silicone is Newtonian with a viscosity of 4.8 × 10^4^ Pa·s. The pink putty has a more complex power-law behaviour but it approximates a Newtonian rheology with a viscosity of 6.2 × 10^5^ Pa·s at strain rates lower than 10^−3^ s^−1^, which are similar to those occurring in our models^49^. The Hikurangi plateau layer has a density of 1,120 kg·m^−3^, which corresponds to a density contrast of 300 kg·m^−3^ in relation to the glucose syrup, and reflects an upper mantle-oceanic plateau density contrast as estimated in nature with an average oceanic crust density of 2,900 kg·m^−3^ ^50^. The Chatham rise, a continental fragment, is also simulated using a mixture of Wacker silicone and pink putty but in weight proportion of 72.25% and 27.75%, respectively. The Chatham rise layer has a density of 1,020 kg·m^−3^, which corresponds to a density contrast of 400 kg·m^−3^ in relation to the glucose syrup, and reflects an upper mantle-Chatham rise density contrast as estimated in nature with an average continental crust density of 2,800 kg·m^−3^ ^51^.

To quantify the upper mantle flow produced by slab sinking, we use a stereoscopic Particle Image Velocimetry (sPIV) technique that allows to compute the 3 components (3 C) of the mantle flow velocity field in a plane. The glucose syrup layer simulating the upper mantle is randomly filled with 20–50 μm diameter polymethylmethacrylate-rhodamine B phosphorescent particles that are illuminated with a horizontal laser sheet at 5.5 cm depth (275 km). More details on the sPIV method applied to subduction models can be found in a previous publication^23^. Two high-resolution cameras (2,046 × 2,046 pixels) placed above the model allow to compute the mantle flow velocity field in map view. The 3 C mantle flow velocity field was computed with a spatial resolution of 1.75 pixels/mm, a seeding density of ~40 particles/cm^2^, an interrogation window of 64 × 64 pixels with an overlap of 50%, and a time lapse between two images of 45 s. After computation and post-processing, a few artefacts remain on the vertical component of the 3 C velocity field because the sPIV system may detect motion of particles other than the phosphorescent markers, such as tiny bubbles anywhere in the glucose syrup. These artefacts cannot be removed as they have the same magnitude as the signal we want to image, but they are easy to distinguish from it as they are of smaller scale than, for instance, the broad scale upwellings (Fig. 3). These artefacts should not be taken into account when interpreting the mantle flow velocity field images. Two other cameras allow to take pictures from one side, allowing to follow the slab geometry in the north, and from the front (mantle wedge) side, allowing to see the slab geometry along the trench. An additional camera placed above the model allows to take pictures when the laser does not emit, to record pictures in map view where passive tracers placed on the plate are seen. The subducting plate velocity is measured using passive markers equidistantly placed both on top of and at the sides of the subducting plate. The trench retreat velocity is measured using the colour contrast between the glucose syrup and the subducting plate.

We performed a set of 10 experiments where we varied the thickness of the Hikurangi plateau and Chatham rise, and also compared results with models that do not include them. Some experiments were repeated to ensure reproducibility (see Table T1 in Supplementary Information). We then selected one model that best reproduces the kinematics of trench motion and slab geometry at depth of the Tonga-Kermadec-Hikurangi subduction zone, to discuss results focusing on the subduction-induced mantle flow around the northern lateral slab edge.

### Tectonic reconstruction

The tectonic reconstruction maps (Figs 1b–d, 5 and Fig. S1 in Supplementary Information) were performed using the GPlates software (version 2.0, http://www.gplates.org). A data bundle provided with the software allowed us to map the TKH trench, the Pacific subducting plate, the Samoan volcanoes, the Hikurangi Plateau, and the Chatham rise^52–55^. A data bundle was also used^1,56–58^, from which the location of the present-day Samoan hotspot was determined. The predicted position of the above geological features back in time was then reconstructed using the global rotation model of Matthews *et al*.^52^ and the Indo-Atlantic moving hotspot reference frame as a reference^59^. In this reference frame, the Samoan hotspot moves slowly southward between 10–0 Ma (<0.65°, Fig. 1c) and in the east-west direction between 14–10 Ma (<0.4°).

### Data Availability

The datasets generated and analysed during the current study are available from the corresponding author on reasonable request.

## Electronic supplementary material

Supplementary Information

## References

[1] Montelli R (2004). Finite-Frequency Tomography Reveals a Variety of Plumes in the Mantle. Science..

[2] Chang S-J, Ferreira AMG, Faccenda M (2016). Upper- and mid-mantle interaction between the Samoan plume and the Tonga-Kermadec slabs. Nat. Commun..

[3] Courtillot V, Davaille A, Besse J, Stock J (2003). Three distinct types of hotspots in the Earth’s mantle. Earth Planet. Sci. Lett..

[4] Morgan WJ (1972). Deep Mantle Convection Plumes and Plate Motions. The American Association of Petroleum Geologists Bulletin.

[5] Hart SR (2004). Genesis of the Western Samoa seamount province: Age, geochemical fingerprint and tectonics. Earth Planet. Sci. Lett..

[6] Koppers AAP (2008). Samoa reinstated as a primary hotspot trail. Geology.

[7] Jackson, M. G. *et al*. Samoan hot spot track on a ‘hot spot highway’: Implications for mantle plumes and a deep Samoan mantle source. *Geochemistry*, *Geophys*. *Geosystems* **11**, Q12009 (2010).

[8] Koppers, A. A. P. *et al*. Age systematics of two young en echelon Samoan volcanic trails. *Geochemistry*, *Geophys*. *Geosystems* **12**, Q07025 (2011).

[9] Natland JH (1980). The progression of volcanism in the Samoan linear volcanic chain. American Journal of Science.

[10] Gurriet P (1987). A thermal model for the origin of post-erosional alkalic lava, Hawaii. Earth Planet. Sci. Lett..

[11] Hawkins JW, Natland JH (1975). Nephelinites and basanites of the Samoan linear volcanic chain: Their possible tectonic significance. Earth Planet. Sci. Lett..

[12] Kincaid C, Griffiths RW (2003). Laboratory models of the thermal evolution of the mantle during rollback subduction. Nature.

[13] Schellart WP (2004). Kinematics of subduction and subduction-induced flow in the upper mantle. J. Geophys. Res. B Solid Earth.

[14] Li ZH, Ribe NM (2012). Dynamics of free subduction from 3-D boundary element modeling. J. Geophys. Res. Solid Earth.

[15] Funiciello F (2006). Mapping mantle flow during retreating subduction: Laboratory models analyzed by feature tracking. J. Geophys. Res. Solid Earth.

[16] Turner S, Hawkesworth C (1998). Using geochemistry to map mantle flow beneath the Lau Basin. Geology.

[17] Smith GP (2001). A Complex Pattern of Mantle Flow in the Lau Backarc. Science..

[18] Jadamec MA, Billen MI (2010). Reconciling surface plate motions with rapid three-dimensional mantle flow around a slab edge. Nature.

[19] Schellart WP (2010). Mount Etna-Iblean volcanism caused by rollback-induced upper mantle upwelling around the Ionian slab edge: An alternative to the plume model. Geology.

[20] Strak, V. & Schellart, W. P. Evolution of 3-D subduction-induced mantle flow around lateral slab edges in analogue models of free subduction analysed by stereoscopic particle image velocimetry technique. *Earth Planet*. *Sci*. *Lett*. **403**, 368–379 (2014).

[21] Schellart WP, Spakman W (2012). Mantle constraints on the plate tectonic evolution of the Tonga–Kermadec–Hikurangi subduction zone and the South Fiji Basin region. Aust. J. Earth Sci..

[22] Fukao Y, Obayashi M (2013). Subducted slabs stagnant above, penetrating through, and trapped below the 660 km discontinuity. J. Geophys. Res. Solid Earth.

[23] Strak, V. & Schellart, W. P. Control of slab width on subduction-induced upper mantle flow and associated upwellings: Insights from analog models. *J*. *Geophys*. *Res*. *Solid Earth* **121**, 4641–4654 (2016).

[24] Mckenzie D, Bickle MJ (1988). The volume and composition of melt generated by extension of the lithosphere. J. Petrol..

[25] Asimow PD, Hirschmann MM, Stolper EM (2001). Calculation of Peridotite Partial Melting from Thermodynamic Models of Minerals and Melts, IV. Adiabatic Decompression and the Composition and Mean Properties of Mid-ocean Ridge Basalts. J. Petrol..

[26] Schmerr N (2012). The Gutenberg Discontinuity: Melt at the Lithosphere-Asthenosphere Boundary. Science..

[27] Herzberg, C. *et al*. Temperatures in ambient mantle and plumes: Constraints from basalts, picrites, and komatiites. *Geochemistry*, *Geophys*. *Geosystems* **8**, Q02006 (2007).

[28] Herzberg, C. & Asimow, P. D. Petrology of some oceanic island basalts: PRIMELT2.XLS software for primary magma calculation. *Geochemistry*, *Geophys*. *Geosystems* **9**, Q09001 (2008).

[29] Hole MJ (2015). The generation of continental flood basalts by decompression melting of internally heated mantle. Geology.

[30] Ribe NM (1985). The generation and composition of partial melts in the earth’s mantle. Earth Planet. Sci. Lett..

[31] Falloon TJ (2007). Boninites and Adakites from the Northern Termination of the Tonga Trench: Implications for Adakite Petrogenesis. J. Petrol..

[32] Foley BJ, Long MD (2011). Upper and mid-mantle anisotropy beneath the Tonga slab. Geophys. Res. Lett..

[33] Meriaux CA (2016). Mantle plumes in the vicinity of subduction zones. Earth Planet. Sci. Lett..

[34] Druken KA, Kincaid C, Griffiths RW, Stegman DR, Hart SR (2014). Plume-slab interaction: The Samoa-Tonga system. Phys. Earth Planet. Inter..

[35] Ritsema J, Deuss A, Van Heijst HJ, Woodhouse JH (2011). S40RTS: A degree-40 shear-velocity model for the mantle from new Rayleigh wave dispersion, teleseismic traveltime and normal-mode splitting function measurements. Geophys. J. Int..

[36] French SW, Romanowicz BA (2014). Whole-mantle radially anisotropic shear velocity structure from spectral-element waveform tomography. Geophys. J. Int..

[37] Ballmer MD, Ito G, van Hunen J, Tackley PJ (2011). Spatial and temporal variability in Hawaiian hotspot volcanism induced by small-scale convection. Nat. Geosci..

[38] Bredow E, Steinberger B, Gassmöller R, Dannberg J (2017). How plume-ridge interaction shapes the crustal thickness pattern of the Réunion hotspot track. Geochemistry, Geophys. Geosystems.

[39] Gassmöller R, Dannberg J, Bredow E, Steinberger B, Torsvik TH (2016). Major influence of plume-ridge interaction, lithosphere thickness variations, and global mantle flow on hotspot volcanism - The example of Tristan. Geochemistry, Geophys. Geosystems.

[40] Jacoby WR (1973). Model experiment of plate movements. Nature.

[41] Schellart WP, Strak V (2016). A review of analogue modelling of geodynamic processes: Approaches, scaling, materials and quantification, with an application to subduction experiments. J. Geodyn..

[42] Duarte JC, Schellart WP, Cruden AR (2013). Three-dimensional dynamic laboratory models of subduction with an overriding plate and variable interplate rheology. Geophys. J. Int..

[43] Schellart, W. P. Kinematics and flow patterns in deep mantle and upper mantle subduction models: Influence of the mantle depth and slab to mantle viscosity ratio. *Geochemistry*, *Geophys*. *Geosystems* **9**, Q03014 (2008).

[44] Ranalli, G. *Rheology of the Earth*. (Chapman and Hall, London, 1995).

[45] Müller RD, Sdrolias M, Gaina C, Roest WR (2008). Age, spreading rates, and spreading asymmetry of the world’s ocean crust. Geochemistry, Geophys. Geosystems.

[46] Korenaga T, Korenaga J (2008). Subsidence of normal oceanic lithosphere, apparent thermal expansivity, and seafloor flattening. Earth Planet. Sci. Lett..

[47] Cloos M (1993). Lithospheric buoyancy and collisional orogenesis: subduction of oceanic plateaus, continental margins, island arcs, spreading ridges, and seamounts. Geol. Soc. Am. Bull..

[48] Mortimer N, Parkinson D (1996). Hikurangi Plateau: A Cretaceous large igneous province in the Pacific. J. Geophys. Res..

[49] Dell’Ertole D, Schellart WP (2013). The development of sheath folds in viscously stratified materials in simple shear conditions: An analogue approach. J. Struct. Geol..

[50] Carlson RL, Raskin GS (1984). Density of the ocean crust. Nature.

[51] Christensen NI, Mooney WD (1995). Seismic velocity structure and composition of the continental crust: A global view. J. Geophys. Res. Solid Earth.

[52] Matthews KJ (2016). Global plate boundary evolution and kinematics since the late Paleozoic: Global and Planetary Change. Glob. Planet. Change.

[53] Müller RD (2016). Ocean Basin Evolution and Global-Scale Plate Reorganization Events Since Pangea Breakup. Annu. Rev. Earth Planet. Sci..

[54] Domeier M, Torsvik TH (2014). Plate tectonics in the late Paleozoic. Geosci. Front..

[55] Gurnis M (2012). Plate tectonic reconstructions with continuously closing plates. Comput. Geosci..

[56] Whittaker JM (2015). Long-term interaction between mid-ocean ridges and mantle plumes. Nat. Geosci..

[57] Steinberger B (2000). Plumes in a convecting mantle: Models and observations for individual hotspots. J. Geophys. Res. Solid Earth.

[58] Anderson, D. L. & Schramm, K. A. In *Special Paper 388: Plates*, *plumes and paradigms* 19–29, (Geological Society of America). 10.1130/0-8137-2388-4.19 (Geological Society of America, 2005).

[59] O’Neill, C., Müller, D. & Steinberger, B. On the uncertainties in hot spot reconstructions and the significance of moving hot spot reference frames. *Geochemistry*, *Geophys*. *Geosystems* **6**, Q04003 (2005).

[60] Wessel P, Smith WHF (1991). Free software helps map and display data. EOS trans. Amer. Geophys. U..

