# Supplementary material for: A subduction and mantle plume origin for Samoan volcanism
Source: Sci Rep. 2018 Jul 11;8:10424. doi: 10.1038/s41598-018-28267-3 (PMC6041271; doi:10.1038/s41598-018-28267-3)
Supplement: Supplementary file 1 — Supplementary Information [file 41598_2018_28267_MOESM1_ESM.pdf]

Supplementary Information to "A subduction and mantle plume origin for Samoan volcanism"

Vincent Strak[1,2,3]*, Wouter P. Schellart[1,2]

[1]School of Earth, Atmosphere and Environment, Monash University, Melbourne, VIC 3800, Australia.

[2]Department of Earth Sciences, Vrije Universiteit Amsterdam, Amsterdam, Netherlands

[3]Instituto Dom Luiz, Lisbon University, Lisbon, Portugal

*Corresponding author emails: vincent.strak@monash.edu, vstrak@fc.ul.pt

*Corresponding author phone (mobile): +33(0) 6 06 46 08 55

In this Supplementary Information we present a table reporting details on the set of models performed (Table S1) and a figure showing the detailed tectonic reconstruction (Fig. S1).

Model #	SP lithospheric thickness (cm)	Crustal Thickness (cm)		η (Pa•s)	Comments/Observations
		HP	CR	Mantle	
1	2	None	None	210	Symmetrical. Slab folding at both lateral edges but not in the centre. It has an effect on trench curvature, which takes a long time to attain a convex shape towards the mantle wedge in the centre of the subduction zone.
2	2	None	None	199	Slightly asymmetrical with slightly faster trench retreat to the north. Upright anticline along all the downdip tip of the slab. No delay in trench curvature formation.
3	2	0.4	0.6	209	Very asymmetrical with progressive slowdown of trench retreat from north to south and trench advance in the south. Slab folding all along the downdip tip of the slab.
4	2	0.3	0.5	205	Very asymmetrical with progressive slowdown of trench retreat from north to south and trench advance in the south. No slab folding along the downdip tip of the slab.
5	2	0.2	0.4	210	Asymmetry only marked in the south while north of HP retreats more or less uniformly, suggesting local effect to the south due to thinner crustal layers of HP and CR. No slab folding along the downdip tip of the slab.
6	2	0.4	0.6	209	Asymmetry not so well developed north of HP. Slab folding along the downdip tip of the slab in the north, resulting in delayed slab rollback.
7	2	0.4	0.6	205	Similar to model 3.
8	2	0.3	0.5	210	Similar to model 4.
9	2	0.2	0.4	210	Trailing edge of SP attached to acrylic sheet. SP rotation is decreased but SP is stretched inducing an important and unrealistic slab curvature.
10	2 (1.6, 1.4)	0.4	0.6	194	Similar to model 3.

Table S1: Table reporting details on the 10 subduction models performed. SP is the subducting plate (Pacific subducting lithosphere). HP and CR indicate the Hikurangi plateau and Chatham rise, respectively. The experiments were sensitive on the interaction between the downdip tip of the slab and the rigid bottom of the tank. The type of deformation (folding) had to be uniform along the downdip tip of the slab to ensure consistency, as it can delay trench retreat at the surface and thus the similitude with the natural case. The numbers into brackets in column 2 refer to the sub-crustal lithospheric thickness below the Hikurangi plateau and Chatham rise, respectively, in order to make a total lithospheric thickness of 2 cm. The model selected for the manuscript is model #3. Models #3, #6, and #7 had the same setup to test reproducibility. Model #3 compares well with model #7 but model #6 is different because of the sensitivity to the interaction between the slab and rigid bottom of the tank. Model #3 also compares well with models #4 and #8 that have slightly thinner crustal thicknesses for the Hikurangi plateau and Chatham rise. Model #2 is similar to the model with subducting plate width of 72 cm presented in Strak and Schellart (2016).

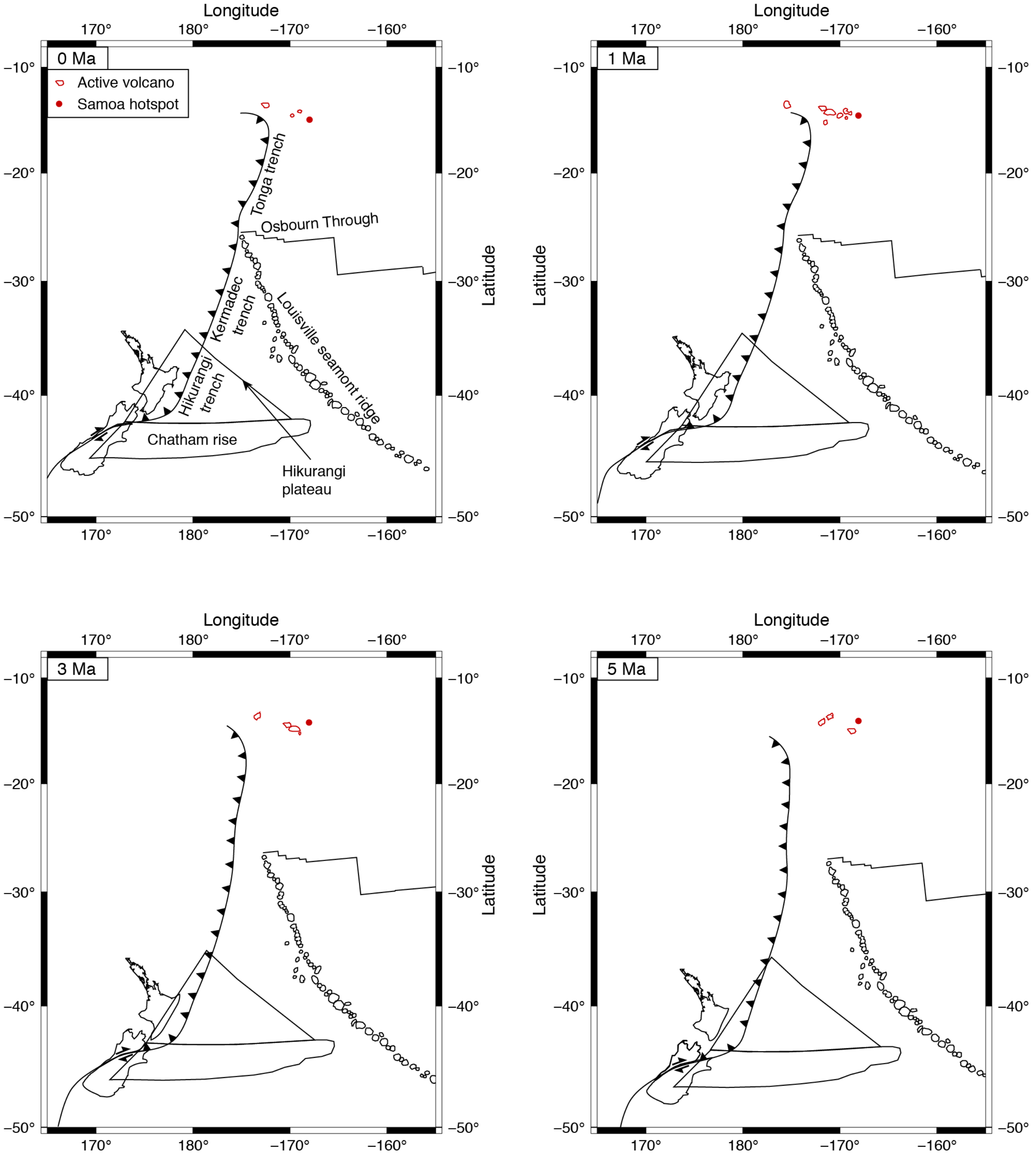

Figure S1: Tectonic reconstruction showing kinematics of the Tonga-Kermadec-Hikurangi trench, Hikurangi plateau, Chatham rise and location of Samoan volcanism and predicted hotspot at 0, 1, 3 and 5 Ma. For detail on how the tectonic reconstruction was produced please refer to the legend of Fig. 1 and the Methods section. The maps were plotted using the GMT software developed by Wessel and Smith (1991) [60] (version 4.5.14, http://gmt.soest.hawaii.edu).

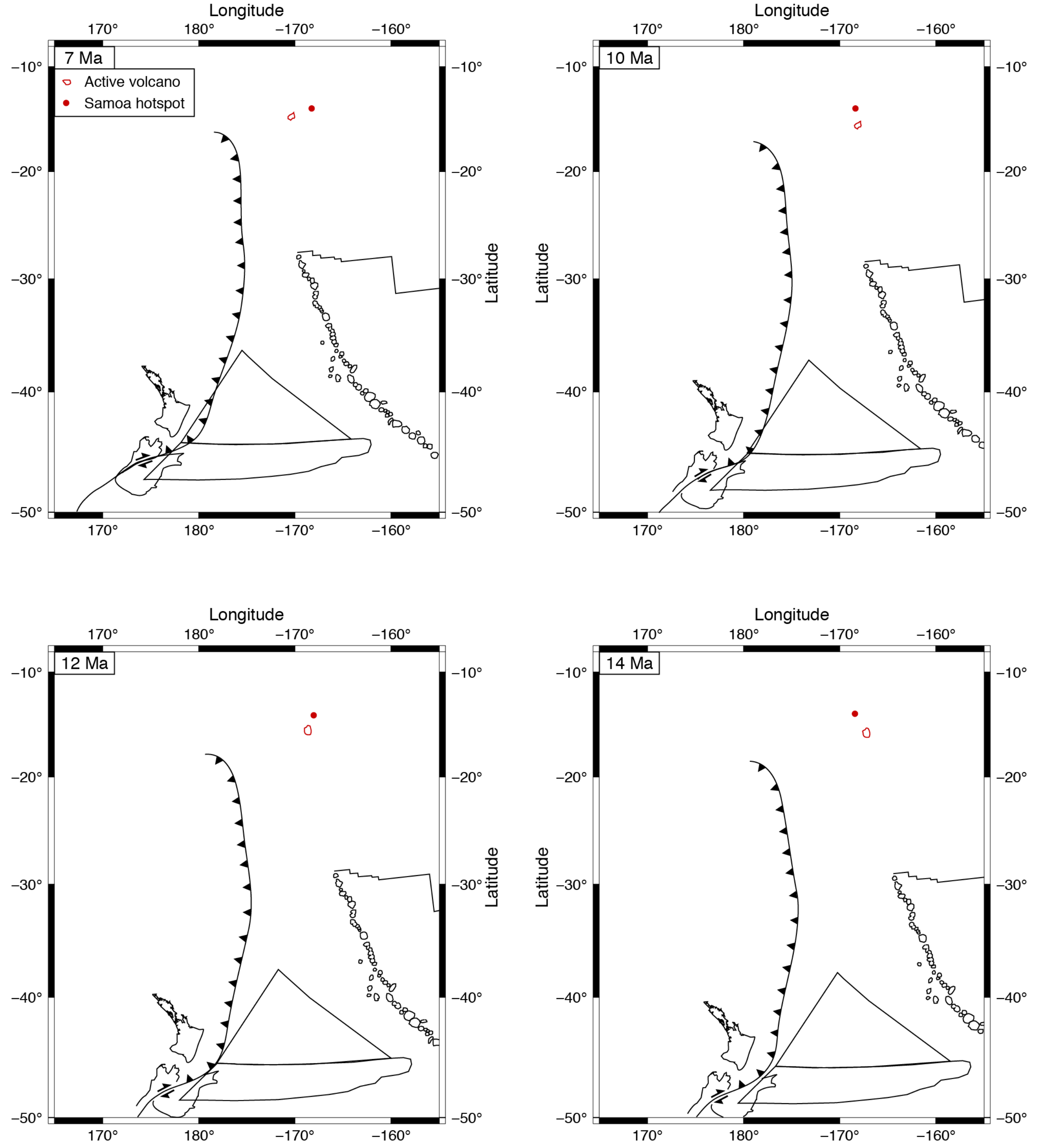

Figure S1 (continued): Tectonic reconstruction showing kinematics of the Tonga-Kermadec-Hikurangi trench, Hikurangi plateau, Chatham rise and location of Samoan volcanism and predicted hotspot at 7, 10, 12 and 14 Ma.
